# Interaction of diosmetin, diosmin and diosmetin-7-O-glucoside with human erythrocytes, their model membrane, hemoglobin and redox-active metal ions

**DOI:** 10.3389/fmolb.2026.1814932

**Published:** 2026-06-15

**Authors:** Teresa Kaźmierczak, Zuzana Lomozová, Přemysl Mladěnka, Marlena Gąsior-Głogowska, Dorota Bonarska-Kujawa, Sylwia Cyboran-Mikołajczyk

**Affiliations:** 1 Department of Physics and Biophysics, Faculty of Biotechnology and Food Science, Wrocław University of Environmental and Life Sciences, Wrocław, Poland; 2 Department of Pharmacognosy and Pharmaceutical Botany, Faculty of Pharmacy in Hradec Králové, Charles University, Hradec Králové, Czechia; 3 Department of Pharmacology and Toxicology, Faculty of Pharmacy in Hradec Králové, Charles University, Hradec Králové, Czechia; 4 Department of Biomedical Engineering, Faculty of Fundamental Problems of Technology, Wrocław University of Science and Technology, Wrocław, Poland

**Keywords:** antioxidant activity, fluorimetry, lipid membrane, lipooxidase, metal chelation and reduction, spectrophotometry, α-glucosidase

## Abstract

Diosmin, a flavonoid with documented vascular-protective properties, together with the closely related compounds diosmetin and diosmetin-7-O-glucoside, has not previously been subjected to a systematic comparative biophysical characterization. In this study, we performed an integrated biophysical comparison of these three structurally related flavonoids to delineate how structural differences translate into distinct patterns of metal-ion coordination, membrane interaction and hemoglobin engagement, thereby providing a mechanistic context for their behavior in complex erythrocyte- and simplified hemoglobin-based redox systems. All three compounds exhibited low direct radical-scavenging activity in the DPPH• assay and showed differences in predicted lipophilicity and metal-ion interactions. Diosmetin, the most lipophilic derivative, selectively chelated Cu^2+^/Cu^+^, while diosmin showed the strongest chelation of both Fe^2+^ and Fe^3+^; none of the tested flavonoids reduced iron ions. Diosmetin inhibited rat intestinal α-glucosidase, and both diosmetin and diosmin inhibited lipoxygenase with comparable potency. In intact human erythrocytes, all flavonoids did not cause hemolysis and did not alter osmotic resistance within the concentration range tested, but they differentially affected cell morphology and response to Fe^3+^-induced oxidative stress. Diosmin and diosmetin-7-O-glucoside predominantly induced echinocyte formation, whereas diosmetin favored stomatocytes and, at the highest concentration, partially reduced Fe^3+^-induced hemolysis. Measurements in erythrocyte-mimetic liposomes showed no effect of any compound on acyl chain order, while diosmetin induced a pronounced decrease in membrane dipole potential, in contrast to the modest changes observed for diosmin, indicating different modes of interaction with the lipid headgroup region. All three flavonoids quenched the hemoglobin tryptophan fluorescence via a static mechanism, with a similar quenching efficiency but differing binding constants. ATR-FTIR spectroscopy did not reveal detectable alterations in the overall secondary-structure content of hemoglobin. Collectively, the results demonstrate systematic differences among diosmetin, diosmetin-7-O-glucoside and diosmin in metal-ion coordination, membrane-related effects and hemoglobin interactions, providing a comparative biophysical basis for distinguishing their activities. Based on these results, diosmetin appears to be a particularly interesting candidate for further biophysical and functional investigation.

## 1 Introduction

Human erythrocytes (RBCs) and hemoglobin (Hb) constitute a critical biophysical interface in the circulatory system, where they are continuously exposed to both endogenous and exogenous bioactive compounds (Mohanty et al., 2014). Although RBCs possess robust antioxidant defense systems, these mechanisms can be overwhelmed by pro-oxidative stressors, particularly redox-active metal ions such as copper (Cu^2+^) and iron (Fe^3+^). These transition metals participate in Fenton-type reactions that generate reactive oxygen species (ROS), leading to hemoglobin oxidation, membrane destabilization and, ultimately, to processes associated with cardiovascular pathologies, including atherosclerosis (Jomova and Valko, 2011). Because RBCs are abundant, anucleate, and possess a well-characterized membrane and cytosolic environment, they provide a convenient and physiologically relevant model for studying how small molecules modulate redox processes, interactions with redox-active metal ions, and membrane integrity at the blood-cell interface (Podsiedlik et al., 2020).

Flavonoids are recognized as nature-derived compounds with beneficial cardiovascular effects, but their biological activity cannot be adequately described by simple *in vitro* antioxidant assays alone (Mladěnka et al., 2010). Instead, their effects are strongly dependent on molecular architecture, which determines not only radical-scavenging capacity but also interactions with lipid bilayers, membrane-associated as well as soluble proteins such as hemoglobin. Importantly, the redox behaviour of flavonoids is also influenced by their concentration, the concentrations of free transition metals and the surrounding cellular and extracellular environment. Under certain conditions, particularly at higher concentrations or in the presence of transition metal ions such as Fe^3+^ or Cu^2+^, flavonoids may exhibit pro-oxidant properties due to redox cycling and metal-ion interactions. *In vivo*, the erythrocyte membrane is a key determinant of red blood cell deformability, stability, and survival in the circulation. Changes in its physical properties, such as fluidity, packing of lipid headgroups and dipole potential, can markedly influence erythrocyte function and susceptibility to oxidative damage. Hemoglobin constitutes a second major target at the blood-cell interface: its interaction with low-molecular-weight ligands involves specific hydrophobic pockets in the α- and β-subunits, where the ligand size, shape, and lipophilicity determine binding behavior and influence the local protein environment (Albani et al., 1985). Therefore, minor structural differences among flavonoids, including the presence, absence, or type of sugar moiety, are expected to modulate membrane association, hemoglobin interactions, and interactions with redox-active metal ions, and thereby influence their protective properties under metal-driven oxidative conditions.

Diosmin (3′,5,7-trihydroxy-4′-methoxyflavone 7-rutinoside) is a flavonoid with documented vascular-protective properties, widely used in vasoprotective therapy (Gouda and Babiker, 2020). For example, diosmin normalized lipid and glycoprotein levels in the blood of hyperlipidemic patients (Huwait and Mobashir, 2022), reduced deoxycorticosterone acetate (DOCA) and high-salt (NaCl) diet-induced hypertension in rats (Silambarasan and Raja, 2012), and alleviated lipid peroxidation while restoring antioxidant levels in the human kidneys and heart (Huwait and Mobashir, 2022). However, its biological activity is closely related to metabolic conversion to its aglycone diosmetin (5,7,3′-trihydroxy-4′-methoxyflavone). This flavonoid has been shown to reduce high blood pressure in pigs (Ahmad et al., 2020), decrease plasma levels of malondialdehyde, increase catalase and superoxide dismutase activities in a hypertensive rat model (Meephat et al., 2021), and have a protective effect against myocardial hypertrophy injuries (Sun et al., 2017). Another derivative of diosmin, diosmetin-7-O-glucoside (3′,5-dihydroxy-7-(beta-D-glucopyranosyloxy)-4′-methoxyflavone), bearing only a glucose moiety, has been poorly described in the literature in terms of its protective activity on the cardiovascular system. However, diosmetin-7-O-glucoside has been reported to prevent cardiac fibrosis (Wang et al., 2023) and to inhibit arachidonic acid-induced platelet aggregation (Lin et al., 2024).

In terms of direct interactions of diosmetin, diosmin, and diosmetin-7-O-glucoside with RBCs, experimental models and/or isolated membranes, and Hb, there is a little information. Only, in terms of membranes, Villa et al. showed that diosmetin prevents the oxidative damage to the membrane of cultured rat hepatocytes, which was attributed to the potential modifications of membrane fluidity leading to its stabilization (Villa et al., 1992). However, flavonoids can also modulate enzymes involved in metabolic and inflammatory components of atherosclerosis, such as α-glucosidase, involved in intestinal carbohydrate digestion and metabolic stress (Chaisin et al., 2021) and lipoxygenase (LOX), which catalyzes the oxidation of polyunsaturated fatty acids and contributes to pro-inflammatory leukotriene production (Cathcart and Folcik, 2000). Therefore, evaluating the effects of the tested compounds on these enzymes provides complementary information about their potential biological and anti-atherosclerotic activity. Only one report showed that diosmetin possesses low inhibitory activity towards LOX (Sroka et al., 2017). To conclude, a systematic biophysical comparison of diosmin, diosmetin and diosmetin-7-O-glucoside that integrates their interactions with redox-active copper and iron ions, human erythrocytes and their membranes, hemoglobin and selected enzymes is currently lacking. The aim of this study was to elucidate how minor structural modifications–specifically the removal or variation of sugar moieties–affect the molecular-level interactions of diosmetin, diosmin, and diosmetin-7-O-glucoside with human erythrocytes, their membranes, hemoglobin and redox-active metal ions. To this end, we employed a set of complementary biophysical methods, including UV–Vis and ATR-FTIR spectroscopy, fluorescence spectroscopy and optical microscopy. These experiments allowed a direct comparison of the three flavonoids with respect to Cu^2+^/Cu^+^ and Fe^2+^/Fe^3+^ chelation and reduction, as well as their effects on erythrocyte morphology, membrane fluidity, and membrane dipole potential, and on hemoglobin interactions.

Regarding the bioavailability of studied compounds, the literature data indicate that diosmin is poorly absorbed in the small intestine (Serra et al., 2008). This compound transits through the small intestine, and in the cecum and colon, under the influence of enzymes of bacterial microflora, it is hydrolyzed to diosmetin and other metabolites (Serra et al., 2008). After oral administration, ∼60% of this compound is absorbed in the form of metabolites, mainly diosmetin (Cova et al., 1992). The plasma concentration of diosmetin, which depending on the form of administration, is in the range of 2–50 ng/mL (Russo et al., 2018). The plasma half-life of diosmetin is relatively long and ranges from 26 to 43 h (Cova et al., 1992), which may result in significantly higher plasma concentrations with continuous supplementation with high doses of this compound (e.g., Daflon®500). In this study, the concentrations of flavonoids used (µM-mM) were selected to enable the detection of subtle biophysical and structural changes in RBCs and Hb that might be undetectable at lower, sub-micromolar doses. While these concentrations may exceed typical steady-state plasma levels, this approach is consistent with established *in vitro* models and provides essential mechanistic insights into molecular interactions under oxidative stress conditions (Cesquini et al., 2003; Dai et al., 2006; Henneberg et al., 2013). Additionally, using rational concentration ratios between the tested flavone and metal might mimic the real situation in much lower concentrations, which are experimentally hardly or, if at all, feasible. Furthermore, in specific contexts such as *α*-glucosidase inhibition, the millimolar concentrations employed are biologically achievable directly within the gastrointestinal tract (Barber et al., 2021). Therefore, the primary focus of this work is the systematic biophysical characterization of direct molecular interactions at the blood-cell interface rather than a pharmacokinetic evaluation of bioavailability.

## 2 Materials and methods

### 2.1 Materials

Diosmetin, diosmin and diosmetin-7-O-glucoside were purchased from ExtraSynthese (Genay Cedex, France). Dimethyl sulfoxide (DMSO), 2,2-diphenyl-1-picrylhydrazyl (DPPH), glutaraldehyde, immersion oil, 1,4-dithiothreitol (DTNB, Ellman’s reagent), 2,2′-azobis (2-methylpropionamidine) dihydrochloride (AAPH), lyophilised human hemoglobin (Hb), bathocuproinedisulfonic acid disodium salt (BCS), 3-(2-pyridyl)-5,6-diphenyl-1,2,4-triazine-4′,4″-disulfonic acid sodium salt (ferrozine), hydroxylamine hydrochloride (HA), CuCl, CuSO_4_·5H_2_O, FeSO_4_·7H_2_O, FeCl_3_·6H_2_O, rat intestinal acetone powder, p-nitrophenyl α-D-glucopyranoside (pNPAG), 4-(2-hydroxyethyl)-1-piperazineethanesulfonic acid (HEPES), HEPES sodium salt, Tween®20, lipoxygenase-V (type I-B from soybean; LOX), linoleic acid (LA), 1,2-di-(9Z-octadecenoyl)-sn-glycero-3-phosphocholine (DOPC), 1,2-di-(9Z-octadecenoyl)-sn-glycero-3-phosphoethanolamine (DOPE), 1-hexadecanoyl-2--(9Z-octadecenoyl)-sn-glycero-3-phospho-L-serine (POPS), cholesterol (Chol), nordihydroguaiaretic acid (NDGA) were obtained from Merck (Darmstadt, Germany). Chemicals used for the preparation of the buffers are: Phosphate Buffer Saline (PBS) tablets (Merck, Darmstadt, Germany), NaCl, (Avantor Performance Materials, Gliwice, Poland), Na_2_HPO_4_·12H_2_O, NaH_2_PO_4_·H_2_O, sodium-EDTA, tris (Chempur, Piekary Śląskie, Poland), KCl, NaOH (“STANLAB” SP. Z O.O., Lublin, Poland), NH_4_Cl, and NaHCO_3_ (EUROCHEM BGD Sp. z o.o., Tarnów, Poland). Methanol, ethanol and chloroform were obtained from “STANLAB” SP. Z O.O. (Lublin, Poland) and trichloroacetic acid (TCA) were purchased from Ubichem Plc (Eastleigh Hampshire, United Kingdom), while FeCl_3_ was purchased from Pol-Aura Sp. z o.o. (Morąg, Zawroty, Poland). Fluorescent probes: 1,6-diphenyl-1,3,5-hexatriene (DPH) and 4-[2-[6-(dioctylamino)-2-naphthalenyl]ethenyl]-1-(3-sulfopropyl)-pyridinium, inner salt (di-8-ANEPPS) were obtained from ThermoFisher Scientific (Thermo Fisher Scientific, Waltham, MA, United States).

### 2.2 Biological material

Whole human blood containing investigated erythrocytes was obtained from the Tadeusz Dorobisz Blood Donation and Treatment Centre in Wrocław as part of a scientific agreement. Consent from a bioethics committee to perform this type of research with blood is not required according to Polish law. The erythrocytes were isolated by centrifugation of the whole blood several times in 0.9% NaCl, 706 xg, 5 min until the supernatant was clear. Prior to the experiments, the erythrocytes were washed in the proper buffer according to the type of experiment (see below) at least three times and diluted to the desired hematocrit used in the experiment.

### 2.3 Methods

#### 2.3.1 Antiradical activity of flavonoids

Flavonoids dissolved in DMSO in desired concentrations were mixed with the stable radical DPPH• dissolved in methanol according to the procedure described previously by Goupy et al. (2003), after minor modifications. Experiments were carried out on a 96-well plate, the final concentration of DPPH• was 200 μM. The absorbance was measured at 517 nm on an Agilent BioTek Epoch Microplate Spectrophotometer (Agilent Technologies, Santa Clara, Canada). The results are presented as the percentage of DPPH• reduction by 200 μM of flavonoids.

#### 2.3.2 Lipophilicity of flavonoids

The lipophilicity of the flavonoids was predicted *in silico* using the SwissADME bioinformatic tool (Daina et al., 2017). Results are presented as consensus logP values.

#### 2.3.3 Inhibition of pro-inflammatory enzymes: rat intestinal α-glucosidase and lipoxygenase (LOX)

Inhibition of rat intestinal α-glucosidase activity by diosmetin was performed according to the procedure described by Koh et al. (2018), with minor modifications. The enzyme was prepared using rat intestinal acetone powder according to the procedure described before by Takenaka and Uchiyama (2001). The enzymatic reaction was performed in a 96-well microplate using a phosphate buffer saline at pH 6.9, and the final volume of the reaction was 250 μL. The concentrations of the tested diosmetin ranged between 1 and 400 μM. The reaction mixture was then incubated for 10 min. Then, pNPAG was added and the kinetics were monitored at 37 °C and λ = 405 nm for 10 min. The control indicated DMSO.

Inhibition of lipoxygenase (LOX) by diosmetin and diosmin was performed according to previously published procedures (Liargkova et al., 2019; Xu et al., 2022), after modification. LOX and linoleic acid (LA) were prepared freshly prior to experiment. The optimal choice of buffers as well as LOX and LA concentrations was first experimentally assessed so that the speed of reaction in control sample (increase in absorbance (A) per minute) was between 0.1 and 0.2. LOX was dissolved in 50 mM Tris-HCl buffer of pH 9 at a concentration of 1 mM and diluted directly before the experiment at 25 μM in the same buffer. LA was prepared as follows: 2 mg of LA, 2 μL Tween®20, 20 μL of 0.1 M NaOH and slow addition of 50 mM Tris-HCl pH 9.0 while mixing to obtain the final concentration of 10 mM. LOX and LA were kept on ice during the experiment. The reaction was carried out in 1 mL in a quartz cuvette and consisted of 100 mM Tris-HCl pH 8.2, 250 nM LOX and DMSO/compound (the final concentration of DMSO was 0.5% v/v). LOX was incubated for 5 min in a buffer and after the addition of the compound the incubation was carried out for 10 min. The reaction was initiated by adding of 200 μM LA to the sample and the same volume of buffer to the blank. The kinetics of the reaction leading to conjugated dienes production was measured for 5 min, at λ = 235 nm at 1 s on double beam spectrophotometer Cary Varian UV-Vis spectrophotometer (Varian, Inc., Palo Alto, CA, United States). The speed of the reaction ΔA/min was calculated on the basis of the normalized kinetic graphs. The enzyme inhibition (%inhibition) was calculated between the first and third minutes with the following equation and the values obtained were used for the calculation of 50% enzyme inhibition (IC_50_):

$$\%inhibition=\frac{{\left(\frac{\Delta A}{\min}\right)}_{\;sample}-{\left(\frac{\Delta A}{\min}\right)}_{DMSO}}{{\left(\frac{\Delta A}{\min}\right)}_{DMSO}\;}\times 100\%$$

#### 2.3.4 Chelation and reduction of the transition metals by flavonoids

Copper (Cu^2+^ and Cu^+^) and iron (Fe^2+^ and Fe^3+^) chelation experiments were performed using BCS and ferrozine assays, respectively. The procedures used were previously described by Říha et al. (2014) and Mladěnka et al. (2011), respectively. The experiments were carried out at pH 7.5 in a 15 mM HEPES buffer. The preparation of the copper/iron ions as well as chelators (BCS and ferrozine) was carried out according to the procedures mentioned. The final concentration of ferrozine and BCS varied from 714 to 1,000 µM based on the type of experiment. The concentration of copper/iron ions was selected so that the ratios of flavonoid:metal ions were between 1:1 and 4:1.

The Cu^2+^ reduction experiment was performed using the same BCS procedure (Říha et al., 2014). The procedure used for Fe^3+^ reduction was previously described by Macáková et al. (2012) and also employed ferrozine. The reaction was performed in a DMSO environment.

The absorbance was read at λ = 483 nm (copper ion experiments) and λ = 562 nm (iron ion experiments) using a microplate reader (Hidex Sense Beta Plus; Hidex Oy, Turku, Finland). In all experiments, as a positive control (100% chelation), HA was used. The preparation of HA was described in the mentioned protocols.

#### 2.3.5 Hemolytic activity of flavonoids and their effect on erythrocyte osmotic resistance

The hemolytic activity of flavonoids and their effect on erythrocyte osmotic resistance were evaluated according to the procedure described by Cyboran et al. (2015). The hematocrit used in the experiments was 1.2% and the reactions were performed in phosphate buffer pH 7.4 (hemolytic assay) or different hypotonic and isotonic NaCl buffers (osmotic resistance assay). The flavonoids concentration in the hemolysis experiment was 50–200 μM, whereas that in the osmotic resistance experiment was 100 μM. For each experiment, incubation of flavonoids with cells lasted 1 h and the temperature was set at 37 °C. Spectrophotometric measurements of hemoglobin released in the supernatant were carried out on Specord 40 (Analytik Jena Gmb, Jena, Germany) at λ = 540 nm. The percentage of hemolysis was counted as the absorbance in the sample divided by the mean absorbance of the 100% hemolysis sample.

#### 2.3.6 Erythrocyte shape changes induced by flavonoids

The experiment was carried out according to the procedure described by Bonarska-Kujawa et al. (2015). Erythrocytes were suspended in 0.9% NaCl and the hematocrit in the samples was 1.2%. The concentration of studied compound used was 100 μM. The shapes were observed under an optical microscope Nikon ECLIPSE E200 (Nikon Europe B.V., Amstelveen, Netherlands) with an attached camera MOTICAM S6 (MoticEurope, S.L.U., Barcelona, Spain) and distinguished based on the Bessis scale (Bessis, 1972) into discocytes, echinocytes, stomatocytes. Cell shapes were observed at 1000x magnification.

#### 2.3.7 Level of reduced glutathione (GSH) in erythrocytes after modification with flavonoids

The experiment was carried out according to the procedure described previously by Kaźmierczak et al. (2025), based on the Ellman’s experimental procedure (Ellman, 1959). To test the impact of compounds on GSH level in RBCs, 2 ml of RBCs (hematocrit 5%) were incubated for 1 h at 37 °C with flavonoids at concentrations ranging from 25 to 100 µM. Moreover, to determine the protective effect of tested flavonoids on GSH in RBCs in oxidative stress conditions, RBCs were also incubated with 50–100 μM of flavonoids following the addition of 60 mM AAPH and 1 h of incubation at 37 °C. After the incubation time, 200 μL of cold 20% TCA was added and samples were centrifuged (2670 xg, 10 min, 3 °C). Next, 1 mL of supernatant was discarded to new test tubes, following the addition of 1 mL phosphate buffer pH 8.2 and 100 μL of 5 mM DTNB. The absorbance of the samples at λ = 412 nm was measured on Specord 40 (Analytik Jena Gmb, Jena, Germany) 30 min after the addition of DTNB.

#### 2.3.8 Inhibition of iron-induced hemolysis by flavonoids

The Fe^3+^-induced hemolysis was performed accordingly to the procedure described before by Kaźmierczak et al. (2025), based on the protocol derived from An et al. (2015). The final concentration of Fe^3+^ in 0.9% NaCl was 250 μM. Before incubation of 2 mL RBCs in 0.9% NaCl (hematocrit 2.4%) with 2 mL of iron ions, the cells were incubated for 1 h with flavonoids at 37 °C. The flavonoid: Fe^3+^ ratios were used in between 1:10 and 6:10. Then, an iron ions solution was added to the samples following the incubation at 37 °C for 1 h. The samples were centrifuged, 15 min, 4 °C, 1363 xg. The absorbance of the released hemoglobin was measured at λ = 412 nm on Specord 40 (Analytik Jena Gmb, Jena, Germany).

#### 2.3.9 Interaction of flavonoids with model erythrocyte lipid membrane

The interaction of diosmetin and diosmin with the model erythrocyte lipid membrane (liposomes) was measured using two fluorescent probes: DPH and di-8-ANEPPS. For both experiments, liposomes with a diameter around 100 nm were created using the following lipids dissolved in chloroform/methanol: DOPC/DOPE/POPS/cholesterol in a molar ratio 55/20/10/15 using the pressure extruder (Lipex®10 mL (EvonikIndustries AG, Birmingham, AL, United States) through Whatman™ Nuclepore™ filters (Cytiva, Global Life Sciences Solutions Poland Sp. z o.o., Kraków, Poland) (Kaźmierczak et al., 2025). The LUVs were prepared in the phosphate buffer pH 7.4. The final concentration of lipids in the sample was 0.25 mM. The experiment with DPH probe was performed according to the procedure described previously (Bonarska-Kujawa et al., 2015). The molar ratio lipids:DPH probe was 100:1. The concentration of flavonoids was between 5 and 20 μM. The anisotropy of the DPH probe was measured in 37 °C on Varian Cary Eclipse spectrofluorimeter (Varian, Inc., Palo Alto, CA, United States). The excitation and emission slits were 5 and 10 nm, respectively. The experiment with di-8-ANEPPS probe was carried out respectively based on the procedure by Cyboran-Mikołajczyk et al. (2017). In this experiment LUVs were prepared in Tris-EDTA pH 7.2, the final concentration of lipids in the samples was 0.25 mM. The molar ratio of lipids to di-8-ANEPPS probe was 400:1. The flavonoids concentrations were 10, 25 and 50 μM. Fluorescence excitation spectra of the probe was measured between 400 and 600 nm using Varian Cary Eclipse spectrofluorimeter (Varian, Inc., Palo Alto, CA, United States) with excitation and emission slits 10 nm. Based on the fluorescence intensity of probe at 420 and 520 nm, the dipole potential (*ψ*_d_) was calculated (Kaźmierczak et al., 2025).

#### 2.3.10 Interaction of flavonoids with human hemoglobin

The concentration of hemoglobin (Hb) was determined spectrophotometrically at room temperature, based on the molar absorption coefficient ε_276_ = 120,808 M^−1^ · cm^−1^ (Chakraborty et al., 2012) and was settled at 3 µM (Das et al., 2018) in phosphate buffer, pH 7.4. The experiment of flavonoids interaction with Hb was carried out on flavonoid titration (1 μL of 5 mM stock solution in DMSO) to 3 mL of hemoglobin. Hb was incubated with the increasing concentration of compound for 3 min, followed by a collection of protein emission spectra excited with wavelengths of λ = 280 nm and λ = 295 nm in the range between 310 and 400 nm. Fluorescence measurements were performed at 37 °C and additionally for diosmetin at 10 °C and 25 °C on Varian Cary Eclipse spectrofluorimeter (Varian, Inc., Palo Alto, CA, United States) with excitation and emission slits of 10 nm. The fluorescence spectra obtained of hemoglobin with flavonoids were corrected for the inner filter effect using the absorbance of hemoglobin with flavonoids at excitation wavelength λ = 295 nm and emission at λ = 330 nm. The following parameters were calculated based on the quenching equations: the Stern-Volmer constant (K_SV_), the bimolecular quenching rate constant (K_q_), the number of binding sites per molecule (n), and the binding constant (K_a_) (Chaudhuri et al., 2011; Chakraborty et al., 2012; Pahari et al., 2013). The τ_0_ – average Hb lifetime without a quencher used was 10^–8^ s (Lakowicz and Weber, 1973).

The interaction of diosmetin with hemoglobin was also checked using Attenuated Total Reflectance–Fourier Transform Infrared Spectroscopy (ATR-FTIR). Hemoglobin was dissolved in phosphate buffer (10 mM, pH 7.4) to a final concentration of 20 mg/mL and incubated for 24 h at 5 °C. Subsequently, the Hb samples were incubated with diosmetin dissolved in DMSO for 1 h. The final concentration of DMSO in all samples was 2% (v/v). The control Hb samples contained the same amount of DMSO. ATR-FTIR spectra were recorded at room temperature using Thermo Fisher Nicolet 6700 FT-IR (Thermo Fisher Scientific, Waltham, MA, United States) equipped with a ZnSe ATR crystal. For each sample, 128 scans at 2 cm^−1^ in a range 4000–700 cm^−1^ were collected and averaged. Spectra of Hb and Hb incubated with diosmetin were processed by subtracting of the corresponding background spectrum of phosphate buffer containing 2% DMSO (using an optimized scaling factor to account for minor variations in ATR film thickness/contact), followed by identical spectral smoothing and baseline correction applied to all spectra. For quantitative comparison, the analysis was focused on the 1800–1100 cm^−1^ region. Spectra were normalized to the Amide I maximum (∼1650 cm^−1^), and an Amide I–normalized difference spectrum was calculated as ΔA = A (Hb + diosmetin) − A (Hb) to visualize subtle changes associated with diosmetin incubation. The spectrum of free diosmetin (recorded and processed under the same conditions) was included in the Supplementary Materials (Supplementary Figure S8) as a reference for band assignment and qualitative assessment of potential ligand contributions; however, it was not subtracted from the Hb + diosmetin spectrum to avoid artefacts due to possible band shifts upon binding. Data processing was performed in OriginPro 2025 (OriginLab Corporation, Northampton, MA, United States), while spectral acquisition and export were carried out using Thermo Scientific EZ OMNIC v. 8.0. The spectrum of phosphate buffer with 2% DMSO was subtracted from each sample using an optimized scaling factor (K) to compensate for minor variations in ATR film thickness/contact. The resulting spectra were smoothed using a Savitzky–Golay filter (17-point window, 3rd-order polynomial) and baseline-corrected using the asymmetric least-squares (AsLS) method. Quantitative comparisons were carried out in the 1800–1100 cm^−1^ region after normalization and difference spectra were calculated as ΔA = A (Hb + diosmetin) − A (Hb).

#### 2.3.11 Statistical analysis

All experiments were performed at least three times. Statistical analyses were performed using PAST 4.16c free software (Hammer et al., 2001). Normality was assessed using the Shapiro-Wilk test. The statistical significances between the control and flavonoid-modified groups in RBCs and model membrane experiments were assessed using t-student test at significance levels p ≤ 0.05 (*), ≤0.01 (**) and ≤0.005 (***). For the statistical significance (p ≤ 0.05) between different experimental groups (DPPH• assay, IC_50_ values in enzymatic experiments, metals chelation and reduction assays) Tukey’s test was applied. Data are presented as mean ± standard deviation (SD).

## 3 Results and discussion

### 3.1 Physicochemical profile of flavonoids

The percentage reduction of DPPH• by the flavonoids tested at 200 μM is presented in Table 1. All compounds exhibited low radical-scavenging activity, which is consistent with their structural characteristics that do not meet the Bors criteria for high antioxidant potency (Bors et al., 1990). Among the analysed compounds, diosmetin showed the highest DPPH• reduction, which may be related to its aglycone character, having one additional free hydroxyl group. Similarly, low antiradical activities have been reported for structurally related flavonoids such as naringenin, naringin, and naringin dihydrochalcone (Kaźmierczak et al., 2025).

**TABLE 1:** Selected physicochemical properties and *in vitro* activities of diosmetin, diosmin, and diosmetin-7-O-glucoside.

Flavonoid	DPPH• reduction, %	Consensus logP	α-glucosidase inhibition, IC_50_, mM	LOX inhibition, IC_50_, μM
*Diosmetin*	12.5 ± 1.2^a^	2.19	0.63 ± 0.03	45.8 ± 3.8^a^
*Diosmin*	6.0 ± 0.5^b^	−0.52	—	48.4 ± 2.7^a^
*Diosmetin-7-O-glucoside*	7.2 ± 1.1^b^	0.57	—	—

^a,b^–values sharing the same indexed letter do not differ significantly at the p ≤ 0.05 level.

DPPH· reduction, % - percentage of DPPH· reduction by diosmetin, diosmin and diosmetin-7-O-glucoside in the concentration of 200 μM. Consensus logP - partition coefficient for the flavonoids derived from SwissADME tool. α-glucosidase inhibition, IC_50_, mM–millimolar concentration of α-glucosidase activity inhibition by 50%. LOX inhibition, IC_50_, μM–micromolar concentration of lipoxygenase (LOX) activity inhibition by 50%.

The SwissADME-predicted consensus logP values are summarized in Table 1 (Daina et al., 2017). Diosmetin showed the highest logP, consistent with predictions reported by Serra et al. (2.69) (Serra et al., 2008), which is plausibly explained by the absence of sugar moieties and thus fewer hydrophilic groups in its structure (Rothwell et al., 2005). In contrast, diosmin, which contains a rutinoside moiety (glucose and rhamnose), exhibited the lowest predicted logP (−0.52), also in agreement with Serra et al. (−0.67) (Serra et al., 2008). Diosmetin-7-O-glucoside was predicted to be more lipophilic than diosmin (0.57), which can be attributed to the presence of a monosaccharide (glucose) rather than the disaccharide substituent in diosmin (Slámová et al., 2018).

Diosmetin, which combined the highest DPPH• reduction and the highest predicted lipophilicity among the tested compounds, was further evaluated for inhibition of rat intestinal α-glucosidase. α-Glucosidase, an intestinal enzyme that breaks down oligosaccharides into glucose, regulates blood sugar and contributes to glycation, RBC damage, and oxidative stress, promoting cardiovascular disease. Diosmetin inhibited the enzyme in a dose-dependent manner (Supplementary Figure S1), with an IC_50_ of ∼0.63 mM (Table 1). Although the IC_50_ is relatively high, it is important to note that α-glucosidase is localized in the gastrointestinal tract, where local concentrations of diosmetin from dietary intake could reach levels sufficient to exert inhibitory effects. The higher micromolar effects of flavonoids such as quercetin or myricetin (above 100 μM) were observed in the intestines (Pico and Martínez, 2019). The presented α-glucosidase inhibiting activity may be related to the presence of free hydroxyl groups in C5 and C7 on the A ring, which have been suggested to facilitate interactions with the enzyme active site (Proença et al., 2017).

Lipooxygenases (LOX) are enzymes that convert fatty acids (e.g., linoleic acid) into leukotrienes and prostaglandins, which trigger pro-inflammatory responses and, if not treated, contribute to tissue damage such as atherosclerosis (Cathcart and Folcik, 2000). Table 1 shows that diosmetin and diosmin inhibited LOX at similar levels; however, both were markedly less potent than the reference inhibitor NDGA (IC_50_ = 1.64 ± 0.33 μM). The inhibition observed in this study was evaluated based on IC_50_ values, which allow comparison of inhibitory potency but do not define the exact mechanism of enzyme inhibition. Previous studies suggest that flavonoids may inhibit LOX through interactions with the enzyme active site, including coordination with the catalytic non-heme iron, or through antioxidant effects that interfere with fatty-acid oxidation (Abeysinghe et al., 1996). In the present study, the weak radical-scavenging activity observed in the DPPH assay (Table 1) indicates that the inhibitory effect of diosmin and diosmetin is unlikely to result solely from general antioxidant activity, which may suggest the contribution of other interaction mechanisms reported for flavonoids. Some studies showed that the glycosides inhibit LOX activity more strongly than their aglycone forms (Sadik et al., 2003; Ha et al., 2010), however, no differences between diosmin and diosmetin were shown in this study. It was reported also previously that diosmetin is a weak lipoxygenase inhibitor in contrast to other flavonoids such as kaempferol or myricetin, which is attributed to the lack of hydroxyl group at C4′ (Sroka et al., 2017). Since both of tested flavonoids differ only with sugar moiety, their similar activity may be attributed to this structural feature.

**FIGURE 1:**
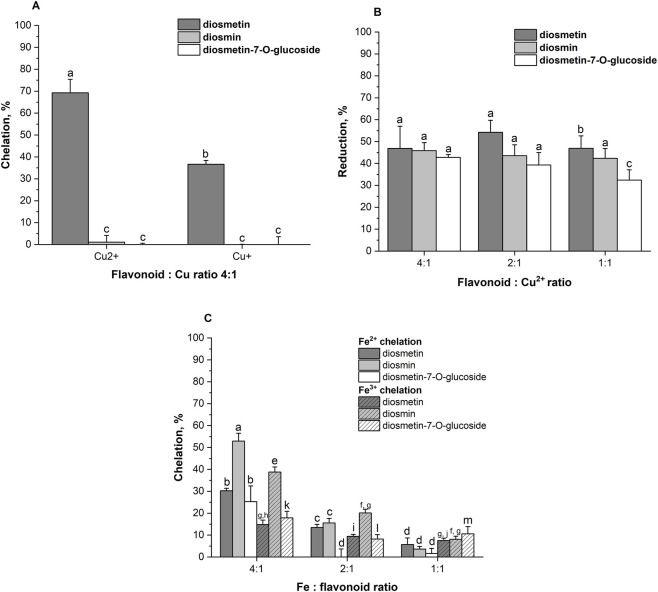
Copper and iron chelation/reduction by diosmetin, diosmin, and diosmetin-7-O-glucoside. **(A)** Cu^2+^ and Cu^+^ (1^+^ and ^+^ in higher index) chelation (%) at different flavonoid: Cu molar ratios. **(B)** Cu^2+^ reduction (%) at different flavonoid: Cu^2+^ molar ratios. **(C)** Fe^2+^ and Fe^3+^ chelation (%) at different flavonoid:Fe molar ratios. Statistical comparisons are indicated by letters and were performed separately for Fe^2+^ (a–d) and Fe^3+^ (e–m). Values sharing the same letter do not differ significantly (p ≤ 0.05).

Copper and iron ions are essential for redox processes and oxygen reactions. However, they also participate in the generation of harmful reactive oxygen species (ROS) via the Fenton reaction (Fe^3+^, Cu^2+^) (Crichton, 2012). Therefore, the reduction and chelation of these ions are essential for redox balance. The flavonoid:metal ratios used in the experiments were limited to a maximum of 4:1, as higher ratios are generally not considered biologically relevant given the low oral bioavailability of flavonoids. Because metal–flavonoid complexation is strongly pH-dependent, the experiments were performed at pH 7.5 to approximate physiological conditions in the bloodstream. Flavonoids, including diosmin, most commonly form complexes with transition metals such as copper and iron with stoichiometries of 1:1 or 2:1, whereas higher-order complexes are strongly dependent on experimental conditions (Catapano et al., 2017; Civa et al., 2019; Lomozová et al., 2021). Only diosmetin chelated Cu^2+^/Cu^+^ at a 4:1 ratio (Figure 1A). Both glycosides (diosmin and diosmetin-7-O-glucoside) were inactive likely due to a hindrance by sugar or modification of electric density. Similarly, baicalein that does not possess a sugar moiety in its structure was shown to have higher chelating activity than its glycoside baicalin (Říha et al., 2014). Diosmetin formed Cu^2+^/Cu^+^ complexes at higher ratios, consistent with Cu^2+^ preferring square-planar or octahedral geometries (Supplementary Figure S2) (Crichton, 2012). In contrast, Cu^+^ prefers a linear geometry, explaining the similar complex yields between ratios of 1.2 and 2. However, hydroxyl groups are less effective donors for Cu^+^ (Crichton, 2012). All flavonoids showed similar copper-reducing activity (Figure 1B), but diosmin and diosmetin-7-O-glucoside reduced Cu^2+^ more strongly, explaining their lack of chelation, in agreement with Lomozová et al. (Lomozová et al., 2022). In harmony with these findings, diosmin moderately reduced Cu^2+^, which is caused by a sugar moiety in the A ring and the methoxy group in the B ring, also present in diosmetin-7-O-glucoside structure (Lomozová et al., 2022). Regarding iron ions, diosmin showed stronger chelating activity at a 4:1 ratio for both Fe^2+^ and Fe^3+^ than diosmetin and diosmetin-7-O-glucoside (Figure 1C). Both Fe^2+^ and Fe^3+^ generally adopt octahedral coordination; there, higher ratios of flavonoid to metal enhance chelation. The higher activity of diosmin may be explained by two factors: 1) iron favours oxygen ligands, and the larger number of potential coordinating groups in diosmin, including the hydroxyls on the flavonoid core and the rutinoside moiety, which can indirectly stabilize the complex—enhances chelation efficiency compared to the other two flavonoids (Walencik et al., 2024); and 2) diosmin, being the most hydrophilic flavonoid (Table 1), is less prone to self-association in aqueous solution, which may increase the accessibility of metal-binding sites and thereby facilitate iron complexation (Slámová et al., 2018). Because diosmetin, diosmin, and diosmetin-7-O-glucoside lack the catechol (3′,4′-dihydroxy) structure in the B ring, iron coordination likely occurs mainly through the 5-OH/4-oxo chelation site of the flavone backbone, while the sugar moiety may influence complex stability indirectly by modifying hydrophilicity and solvation. In this context, the weaker Fe^2+^-chelating activity of diosmetin-7-O-glucoside, despite its slight Fe^3+^-chelating effect, may reflect differences in the ability of the glucose and rutinoside substituents to stabilize the metal complex in aqueous medium rather than the creation of a distinct chelation site. Furthermore, Fe^3+^ is more readily chelated than Fe^2+^ because it preferentially bonds to weaker donors (Slámová et al., 2018; Walencik et al., 2024). Mladěnka et al. also reported the iron-chelating activity of diosmin, showing that at a 10:1 ratio it strongly chelated Fe^2+^, but its activity was much lower at a 1:1 ratio (Mladěnka et al., 2011). Although ratios greater than 4:1 were not tested in the present study, the results for the ratio 1:1 were consistent with these findings. None of the flavonoids showed iron-reducing activity (data not shown). This may be due to: (1) higher chelating activity, which suppresses reduction capacity, and (2) the methoxy group in the B ring, which disfavours reductive activity (Mladěnka et al., 2011).

### 3.2 Interactions of flavonoids with human erythrocytes model lipid membrane

To evaluate the effects of flavonoids tested on RBCs, hemolysis, osmotic resistance, cell morphology, glutathione (GSH) status, and protection against Fe^3+^-induced hemolysis were assessed. Diosmetin-7-O-glucoside did not induce erythrocyte hemolysis within the 5–200 μM range (Supplementary Figure S3A). Diosmetin and diosmin tested at the same concentrations caused only a slight increase in hemolysis; however, the observed values did not exceed 5% to 7% (Supplementary Figure S3A) after 1 h of incubation. Therefore, within the applied conditions, the compounds can therefore be considered non-destructive to the erythrocyte membrane. Moreover, at 100 μM, none of the flavonoids altered the osmotic resistance of the RBCs. The C_50_ values (NaCl concentration causing 50% hemolysis) for flavonoid treated cells were comparable to the control: 0.45% ± 0.01% NaCl (control), 0.45% ± 0.01% NaCl (diosmetin), 0.44% ± 0.02% NaCl (diosmin), and 0.46% ± 0.01% NaCl (diosmetin-7-O-glucoside) (Supplementary Figure S3B).

**FIGURE 2:**
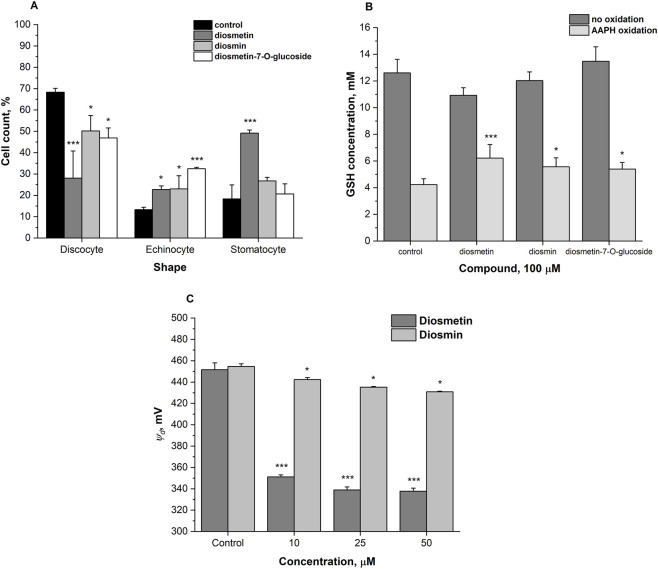
Interaction of diosmetin, diosmin and diosmetin-7-O-glucoside with human erythrocytes (RBCs) and model lipid membrane (liposomes: DOPC/DOPE/POPS/cholesterol). **(A)** Distribution of RBC morphologies in control and flavonoid-treated samples after incubation with 100 μM flavonoids (optical microscopy). **(B)** Reduced glutathione (GSH) levels in untreated RBCs and in RBCs exposed to AAPH after preincubation with 100 μM flavonoids. **(C)** Dipole potential (*ψ*_d_) of liposomes with increasing concentration of flavonoids. Significantly different from control (*-p ≤ 0.05, **-p ≤ 0.01, ***-p ≤ 0.005).

The RBCs provide a convenient model for studying compound–membrane interactions. Their morphology (discocytes, echinocytes, stomatocytes) depends on pH, ionic strength, and the balance between membrane surface area and cell volume (Deuticke, 1968; Bessis, 1972; Stasiuk et al., 2009). The effects of 100 µM flavonoids on RBCs morphology are shown in Figure 2A and Supplementary Figure S4 (microscopic images). Diosmin and diosmetin-7-O-glucoside predominantly induced echinocyte formation relative to the control, whereas diosmetin mainly promoted stomatocyte formation. This pattern is consistent with the predicted lipophilicity of the compounds: diosmetin, with the highest consensus logP (2.19), is expected to integrate into the membrane more readily than diosmin (−0.52) and diosmetin-7-O-glucoside (0.57) (Table 1), which can influence the direction of membrane curvature and therefore, cell shape. Similar effects were observed for cyanidins containing sugar moieties, which promoted echinocyte formation for both rutinoside- and glucose-substituted derivatives (Cyboran-Mikołajczyk et al., 2019).

The flavonoids tested alone did not modify the levels of GSH in RBCs. When the RBCs were treated with AAPH, there was a slight increase in GSH concentration in cells treated with tested flavonoids compared to the control (Figure 2B). Gwozdzinski et al. reported, using the thiol-sensitive fluorescent probe o-phtalane, that diosmin slightly increased GSH and total thiol levels in RBCs under physiological conditions (Gwozdzinski et al., 2023). On the other hand, in the H_2_O_2_-exposed endothelial cells Wójciak et al. demonstrated that 250 μM of diosmetin led to the increased glutathione peroxidase activity, resulting in the protection of these cells against oxidative stress (Wójciak et al., 2022). To date, no data are available on the effects of diosmetin-7-O-glucoside on the GSH levels in RBCs. The percentage inhibition of Fe^3+^-induced hemolysis is presented in Supplementary Figure S3C. Fe^3+^ participates in the Fenton reaction, generating free radicals that can damage cellular barriers and induce hemolysis in RBCs. The radical scavenging activity of compounds can terminate this reaction, thereby protecting biological structures such as cell membranes. Among the tested flavonoids, only diosmetin at the highest concentration (150 μM) showed a statistically significant prevention of Fe^3+^-induced hemolysis, although its protective effect was low (Supplementary Figure S3C). These findings are consistent with the results from the DPPH• assay, in which the flavonoids exhibited low antiradical activity, with diosmetin being slightly more potent (Table 1). While the DPPH• assay does not fully replicate the complex conditions of a biological system and may not directly reflect *in vivo* antioxidant activity, it remains a widely used and valuable method for the rapid *in vitro* assessment of free radical scavenging capacity. Diosmetin’s antioxidant effects on iron-induced stress have already been noted in rat hepatocytes (Morel et al., 1993), but direct RBCs protection by these flavonoids has not been previously reported.

The differential metal-chelating properties and membrane interaction profiles of diosmetin and diosmin suggest that these flavonoids may modulate redox processes in distinct microenvironments under conditions of metal overload. Diosmetin showed stronger interaction with Cu^2+^ ions and a greater ability to penetrate the lipid bilayer, as reflected by its effect on the membrane dipole potential. This behaviour suggests that diosmetin may preferentially influence copper-driven oxidative reactions occurring within the membrane or at the lipid–water interface, where lipid peroxidation processes are initiated. In contrast, diosmin exhibited stronger chelation of Fe^2+^ and Fe^3+^ and is considerably more hydrophilic, which likely limits its penetration into the hydrophobic core of the membrane. Consequently, diosmin may act predominantly in the aqueous phase or at the membrane surface, where it could interfere with iron-mediated redox cycling and Fenton-type reactions. In this context, the two flavonoids may provide complementary modes of redox modulation rather than identical antioxidant functions. Such compartment-dependent activity could be particularly relevant under conditions of metal overload, where oxidative processes occur simultaneously in both membrane and aqueous environments. However, these mechanistic interpretations remain hypothetical and require further verification in biological systems.

In order to delve into the mechanisms of interaction of compounds with the erythrocyte membrane, a model of the erythrocyte membrane (liposomes) was created from lipids naturally occurring in the membrane of these cells in the largest amounts: DOPC, DOPE, POPS and cholesterol (Krivić et al., 2022). The DPH probe integrates deeply into the hydrophobic core of the membrane. The changes in DPH anisotropy give an information about the changes in the acyl chains and potential integration of compounds with hydrophobic parts of the membrane (He, 2023). No changes in anisotropy were observed with the increasing concentrations of the flavonoids (Supplementary Figure S5A), leading to the conclusion that these flavonoids do not interfere deeply into the membrane. Di-8-ANEPPS is an electrochromic fluorescent probe, sensitive to the changes in dipole distribution on the membrane (Cyboran-Mikołajczyk et al., 2017). Excitation spectra of di-8-ANEPPS probe with increasing concentrations of diosmetin and diosmin are shown in Supplementary Figure S5. The spectra with diosmetin are red-shifted (Supplementary Figure S5), while for diosmin no shift of the spectra maximum was observed (Supplementary Figure S5B). The dipole potential (*ψ*_d_) changes for liposomes with increasing concentration of diosmetin and diosmin is shown in Figure 2C. While diosmin led to a small decrease in *ψ*_d_, diosmetin alleviated it strongly. The results showed that both of these flavonoids are capable of interacting with the lipid headgroups and altering the local organization of the dipoles (Efimova and Ostroumova, 2023). However, the mechanisms by which diosmetin and diosmin interact with membranes appear to be different, as indicated by the distinct spectral behaviour of the di-8-ANEPPS probe (Supplementary Figure S5). Diosmetin, which lacks a sugar moiety, reduces the membrane dipole potential markedly, strongly alternating the polar lipid headgroups. It suggests its penetration into the hydrophobic-hydrophilic transition region of the membrane. Diosmin, in contrast, induces only modest changes in dipole potential, consistent with its more superficial interactions with the membrane. These effects correlate well with the erythrocyte shape transformations observed (Figure 2A) and can be interpreted within the framework of bilayer couple theory (Sheetz and Singer, 1974). Diosmetin, due to its higher hydrophobicity (Table 1) induced a strong perturbation in lipid headgroups. It is likely to be intercalated deeper into the membrane. Alternation in the local lipid environment may induce changes in the membrane-associated proteins, resulting in the transformation of the cell shape into both stomatocytes and echinocytes (Lim et al., 2002). Whereas diosmin, with its larger sugar moiety and higher hydrophilicity (Table 1), binds predominantly to the surface of the membrane, leading to the echinocyte formation (Figure 2A). Similar results were obtained for other glycosides, which led to small dipole potential changes such as their aglycones (Efimova and Ostroumova, 2023). No changes in DPH probe anisotropy are also in accordance with previously published results, which showed that flavonoids predominantly localize within the membrane interfacial region, modulating dipole potential and headgroup organization without affecting acyl chain order (Ollila et al., 2002). This mode of interaction also provides a plausible explanation for the observed protection against Fe^3+^-induced hemolysis, as localization of diosmetin within the membrane interfacial region places it in close proximity to sites of iron-driven oxidative reactions and lipid peroxidation, enabling modulation of these processes directly at the membrane level.

### 3.3 Interaction of flavonoids with human hemoglobin

The interactions between flavonoids and human Hb were investigated using fluorescence and ATR-FTIR spectroscopy. Hb shows an emission maximum at 330 nm upon excitation at 295 nm (Supplementary Figure S6), dominated by tryptophan fluorescence. Because some tryptophan (Trp) residues lie near α–β subunit interfaces, including the α_1_β_2_/α_2_β_1_ contact region that undergoes the largest rearrangement during the T↔R transition, ligand binding may alter their microenvironment and thus affect the observed Trp signal (Lakowicz, 2006). In particular, Hb has two putative flavonoid-binding pockets, one in the α chain and one in the β chain. The β-chain pocket is smaller (∼522 Å^3^), typically accommodating smaller flavonoids and is associated with a lower contribution to the Trp fluorescence signal. On the α-chain pocket is larger (∼653 Å^3^) and may accommodate larger flavonoids (Chakraborty et al., 2012). Molecular volumes of studied flavonoids estimated using Molinspiration Cheminformatics (RRID:SCR_018525): 255.81 Å^3^ (diosmetin), 511.79 Å^3^ (diosmin), and 387.93 Å^3^ (diosmetin-7-O-glucoside).

All flavonoids quenched the Hb Trp fluorescence via a static mechanism, as the calculated K_q_ values exceeded 10^10^ M^−1^·s^−1^ (Table 2), consistent with complex formation (Lakowicz and Weber, 1973). The tested compounds showed similar K_SV_ and K_q_ values (Table 2), indicating comparable quenching efficiencies. In contrast, the binding constants (K_a_) followed the trend diosmin ≥ diosmetin ≫ diosmetin-7-O-glucoside (Table 2). Diosmetin and diosmin exhibited an apparent 1:1 binding stoichiometry (n∼1; Table 2), comparable to those reported for 3-hydroxyflavone (Chaudhuri et al., 2011) and quercetin (Gebicka and Banasiak, 2009). Diosmetin-7-O-glucoside displayed a lower apparent stoichiometry (n∼0.75; Table 2), suggesting weaker and/or non-ideal binding behaviour. Overall, these findings indicate similar quenching outcomes accompanied by differences in binding affinity. The observed differences in binding constants may reflect variations in the accessibility of hemoglobin binding cavities. Considering the reported dimensions of the Hb binding pockets and the calculated molecular volumes of the studied flavonoids, larger ligands such as diosmin may preferentially occupy the larger α-chain cavity, whereas smaller molecules such as diosmetin may fit within the smaller β-chain pocket. Despite its intermediate molecular size, diosmetin-7-O-glucoside showed substantially lower Ka and n < 1, suggesting weaker and possibly heterogeneous or multi-site binding to Hb. The hypothesis can be confirmed by the results of binding structurally similar flavonoids such as rutin and quercetin to bovine Hb performed using molecular docking (Das et al., 2018). However, fluorescence quenching alone does not allow definitive assignment of the binding site, and this hypothesis requires further verification using complementary approaches such as competitive binding assays, molecular docking, or site-specific probing experiments.

**TABLE 2:** Biophysical (fluorescence quenching and binding) parameters describing interactions of human hemoglobin with diosmetin, diosmin, and diosmetin-7-O-glucoside.

Flavonoid	K_SV_ (M^−1^)·10^4^	R^2^	K_q_ (M^−1^s^−1^)·10^12^	K_a_ (M^−1^)·10^5^	R^2*^	n
*Diosmetin*	2.85	0.991	2.85	1.23	0.978	1.13
*Diosmin*	3.29	0.988	3.29	2.66	0.996	1.19
*Diosmetin-7-O-glucoside*	3.51	0.987	3.51	0.02	0.988	0.75

K_SV_, stern-volmer quenching constant; K_q_, bimolecular quenching rate constant; K_a_, binding constant; n, number of binding sites per molecule; R^2^, correlation coefficient for K_SV_ and K_q_ values; R^2^*, correlation coefficient of K_a_ and n values. The measurements were performed in 37 °C.

To confirm the static mechanism of binding and better elucidate the binding dynamics of flavonoids, temperature-dependent measurements were performed on diosmetin. This flavonoid was chosen, because the quenching parameters were very similar for each of the three tested flavonoids. The dependence of F_0_/F on the concentration of diosmetin at 10, 25, and 37 °C is presented in Supplementary Figure S7 (Supplementary Material). The linear nature of the plots and the temperature-dependent trend confirm the formation of a stable ground-state complex. Other research also confirms that flavonoid binding to proteins causes static mechanism of fluorescence quenching (Yuan et al., 2008; Zhang et al., 2012; Das et al., 2018).

**FIGURE 3:**
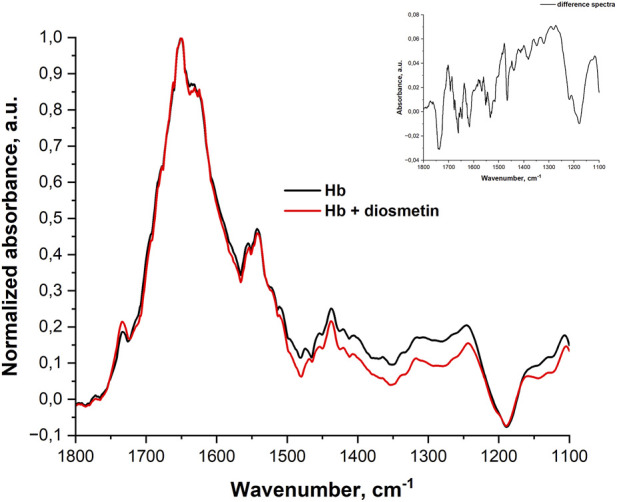
ATR-FTIR spectra of human hemoglobin (Hb; black) in phosphate buffer and the Hb–diosmetin complex (red) in the 1800–1100 cm^−1^ region, normalized to the Amide I maximum (∼1650 cm^−1^). *Inset:* difference spectrum (ΔA = A_complex − A_Hb) calculated after normalization to the amide I band maximum.

Given the comparable static quenching mechanisms and efficiencies shared by the three flavonoids, diosmetin was also selected as a representative model for ATR-FTIR characterization to establish a structural baseline for the flavone-hemoglobin interaction, particularly since this aglycone exhibited the most pronounced biophysical activity across our membrane and enzyme assays. The Amide I band, dominated by peptide backbone C=O stretching vibrations, is widely used as a marker of the protein secondary structure (Ruggeri et al., 2020). In ATR-FTIR measurements, spectra were normalized to the Amide I maximum (∼1650 cm^−1^) to facilitate comparison of backbone-related features (Figure 3). In both Hb and Hb incubated with diosmetin, the characteristic Amide I (∼1650 cm^−1^) and Amide II (∼1550 cm^−1^) bands were observed, together with weaker features in the fingerprint region.

In two independent replicates, the position of the Amide I maximum remained essentially unchanged after incubation with diosmetin (1648.9 ± 1.4 cm^−1^ for Hb and 1648.9 ± 2.7 cm^−1^ for Hb + diosmetin; mean ± SD, n = 2). Normalized spectra were highly similar, indicating no detectable changes in the hemoglobin secondary-structure signature under the applied conditions. Any differences between Hb and Hb + diosmetin were subtle and appeared mainly as low-amplitude intensity variations, most evident in the Amide II and fingerprint regions. Such small changes can arise from localized ligand–protein interactions and microenvironmental effects (e.g., hydration and hydrogen-bonding changes) and may also include weak contributions from diosmetin vibrational modes (Torii and Kawanaka, 2016).

Importantly, these ATR-FTIR observations are consistent with the fluorescence-based results: while fluorimetry indicates diosmetin binding to Hb (static quenching and binding parameters), ATR-FTIR does not show spectral shifts indicative of major conformational rearrangements (Supplementary Figure S8). Overall, the combination of both approaches suggests that diosmetin binds to Hb without appreciable global reorganization of hemoglobin secondary structure and likely induces only subtle, local perturbations. Similar behaviour has been reported for human serum albumin, where diosmetin binding did not measurably alter the protein secondary structure (Zhang et al., 2012).

## 4 Conclusion

This study presents a biophysical characterization of the flavone diosmetin and its glycosides, diosmin and diosmetin-7-O-glucoside, clarifying how subtle structural variations—particularly the presence of different patterns of glycosylation—govern their interactions with redox-active copper and iron ions, RBCs, erythrocyte-mimetic membranes, and human Hb. Diosmetin inhibited α-glucosidase in a dose-dependent manner, while both diosmetin and diosmin suppressed lipoxygenase (LOX) activity with comparable potency, indicating that glycosylation does not substantially impair the active-site interactions required for LOX inhibition. Diosmetin possessed higher lipophilicity and its aglycone form was effective in copper (Cu^2+^/Cu^+^) chelation. On the contrary, the more oxygen ligands and higher hydrophilicity of diosmin led to its higher chelation of iron (Fe^2+^/Fe^3+^). None of the tested flavonoids exhibited iron-reducing activity, suggesting that their protective effects are not mediated by direct reductive mechanisms. Although all compounds were non-destructive to human erythrocytes, their membrane-level modes of action differed substantially. Aglycone diosmetin interacted more strongly with lipid headgroups, significantly lowering the membrane dipole potential and inducing stomatocyte formation. Conversely, the more hydrophilic diosmin localized mostly at the membrane surface and promoted echinocyte formation. Notably, only diosmetin conferred measurable protection against iron-induced hemolysis at the highest flavonoid-to-iron ratio. All three flavonoids formed stable complexes with hemoglobin via a static quenching mechanism. Although the quenching efficiencies were similar, the binding constants followed the trend diosmin ≈ diosmetin ≫ diosmetin-7-O-glucoside, implying that molecular volume and glycosylation pattern influence pocket selectivity. ATR-FTIR analysis of the representative diosmetin–hemoglobin complex further confirmed that binding occurs without inducing global alterations in secondary protein structure.

In summary, diosmetin displays the most favourable overall biophysical profile, integrating strong membrane affinity with effective modulation of metal ions and enzyme activity. Collectively, these findings demonstrate how subtle structural modifications can profoundly reshape flavonoid biophysical behaviour, thereby determining their capacity to protect blood components from metal-driven oxidative and pro-inflammatory stress.

## Data Availability

The datasets generated and analysed during the current study are available in the Base of Knowledge of Wrocław University of Environmental and Life Sciences, http://dx.doi.org/10.57755/jrgr-vv91.
