# Supplementary material for: Interaction of diosmetin, diosmin and diosmetin-7-O-glucoside with human erythrocytes, their model membrane, hemoglobin and redox-active metal ions
Source: Front Mol Biosci. 2026 Jun 15;13:1814932. doi: 10.3389/fmolb.2026.1814932 (PMC13310901; doi:10.3389/fmolb.2026.1814932)
Supplement: Supplementary file 1 [file DataSheet1.docx]

Supplementary Material

## Supplementary Figures


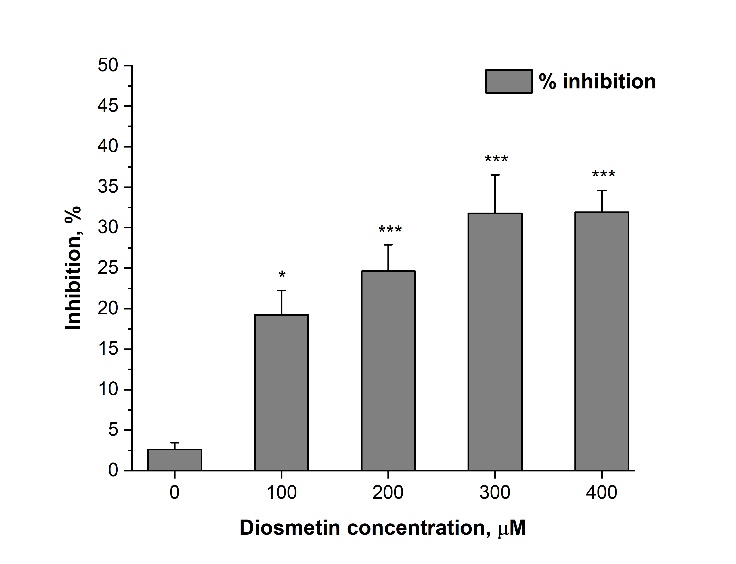


**Supplementary Figure 1.** Percentage of inhibition of the activity of rat intestinal α-glucosidase activity by diosmetin in increasing concentrations. Statistically significant values between control and samples, (*-p ≤ 0.05, **-p≤0.01, ***-p≤0.005). .


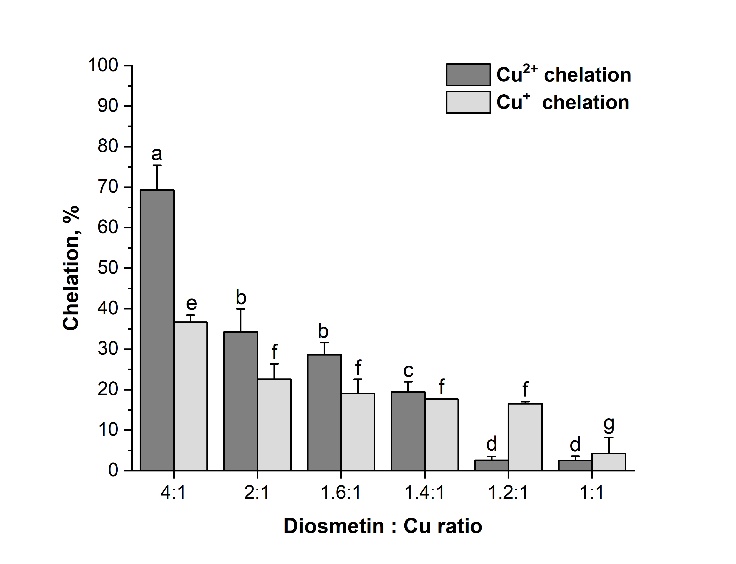


**Supplementary Figure 2.** Cu^2+^ and Cu^+^ chelation by diosmetin in different diosmetin:Cu concentration ratios; presented statistical comparisons between samples as letters were performed separately for Cu^2+^ (a-d) and Cu^+^ ions (e-g) with p≤0.05.


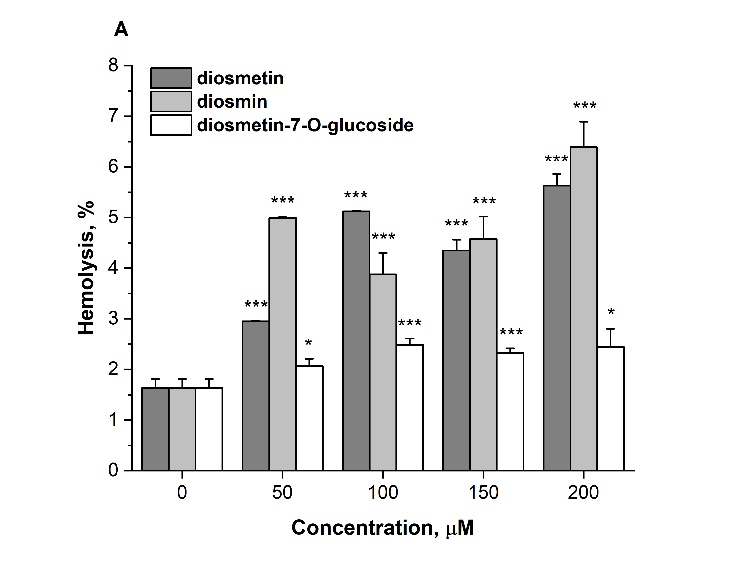

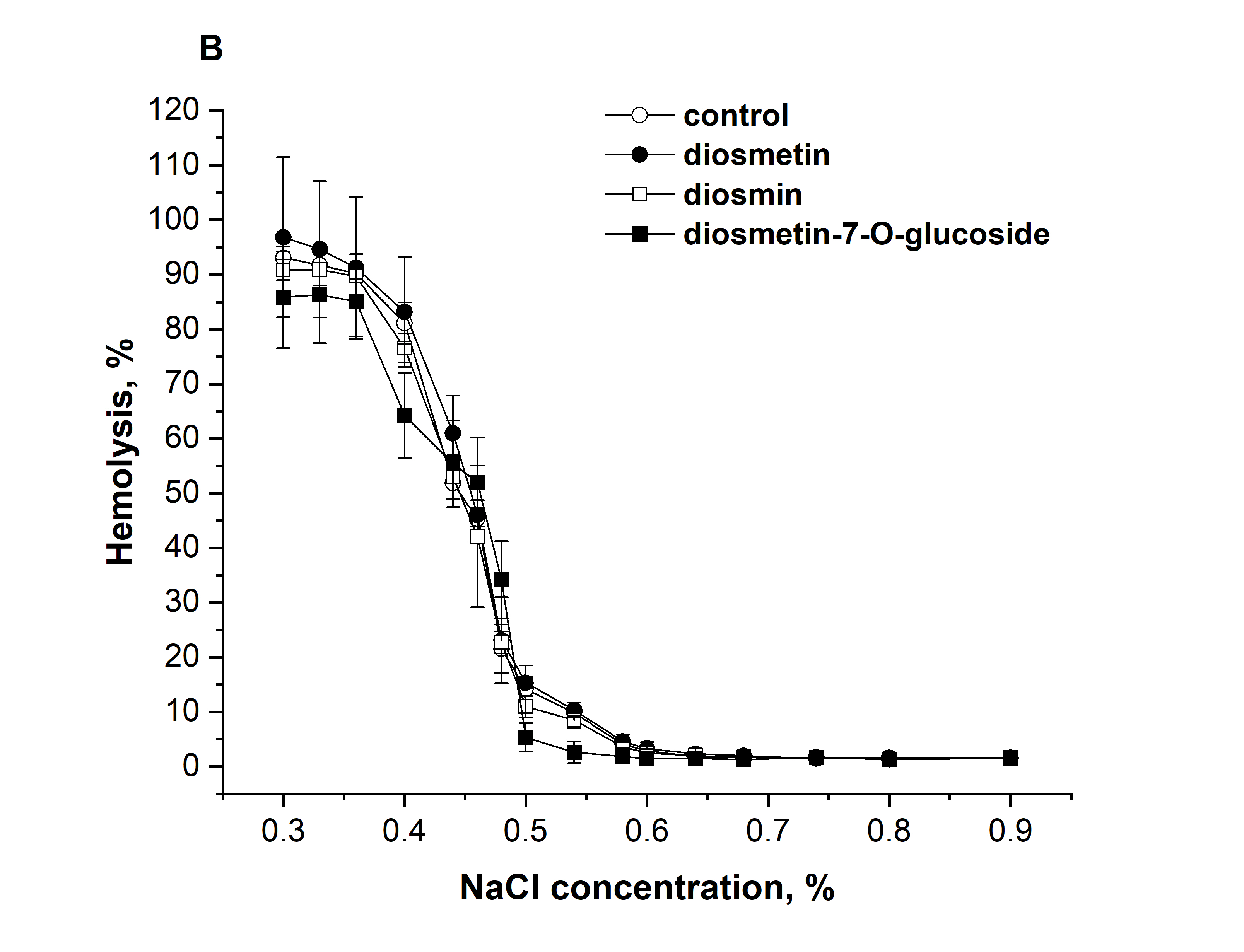

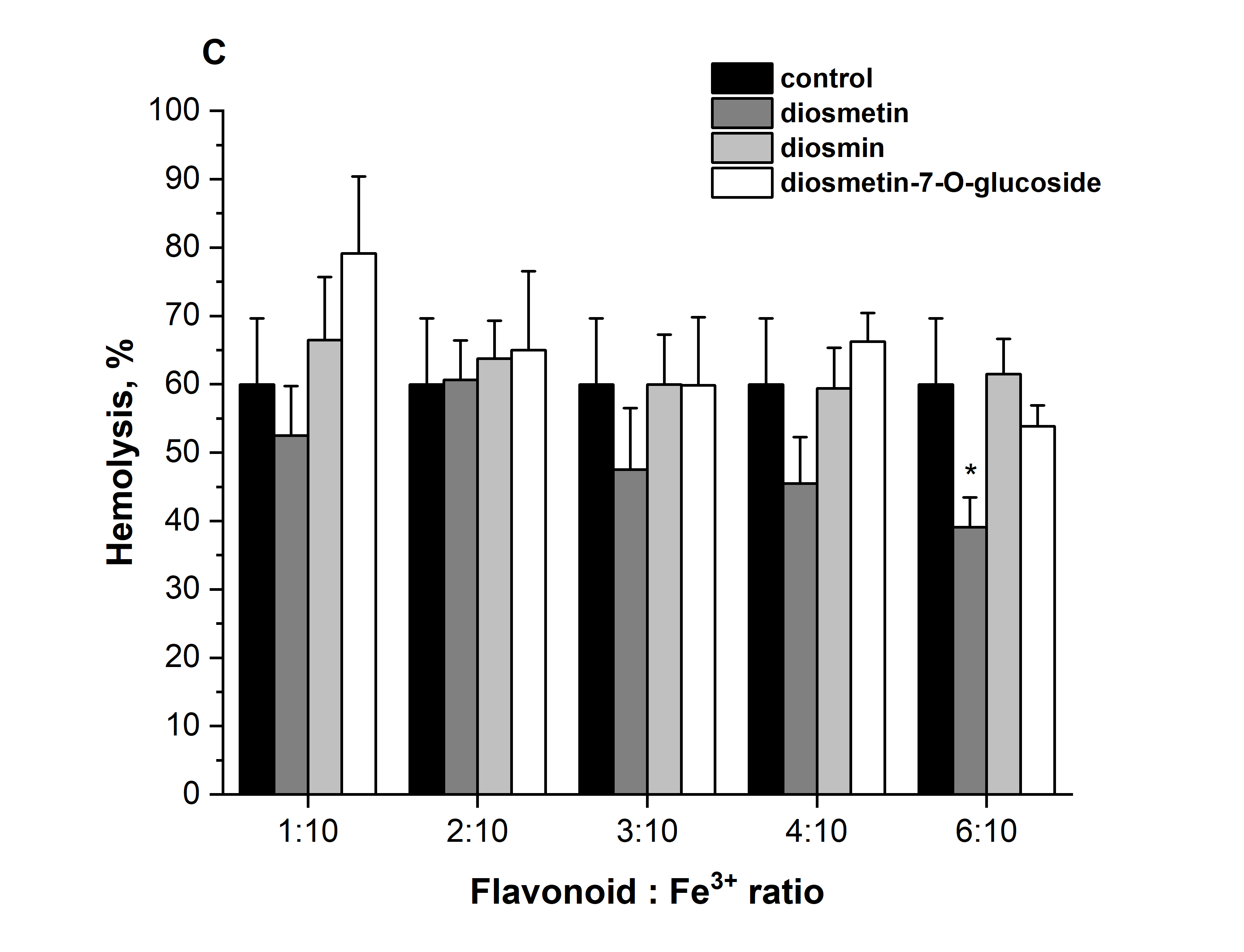


**Supplementary Figure 3.** Interaction of flavonoids with human erythrocytes. (A) Flavonoid-induced hemolysis of RBCs in a concentration range 50-200 μM; (B) Hemolytic curves for control and 100 μM flavonoid-modified RBCs in concentration of 100 μM. (C) Fe³⁺-induced hemolysis (%) at different flavonoid:Fe³⁺ concentration ratios. Statistically significant values between control and samples, (*-p ≤ 0.05, **-p≤0.01, ***-p≤0.005)
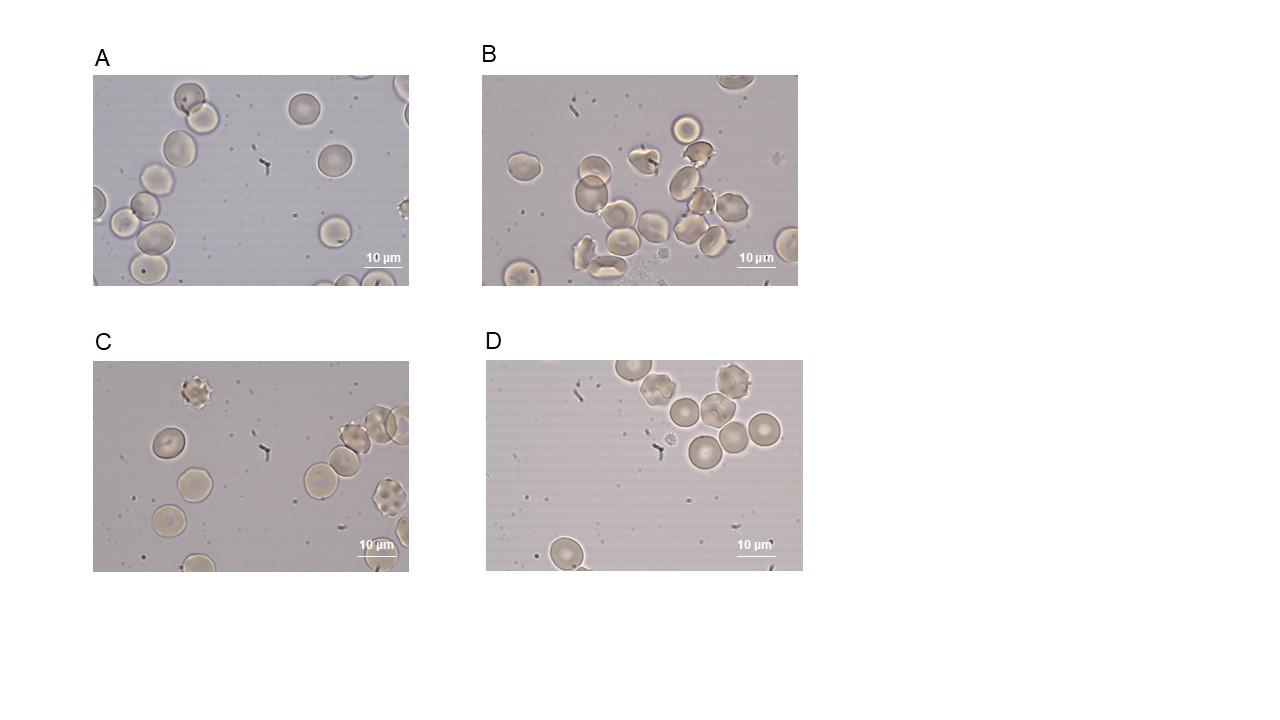


**Supplementary Figure 4.** Shapes of erythrocytes observed under optical microscope with magnitude 1000x: control (A), diosmetin (B), diosmin (C), diosmetin-7-O-glucoside (D). The concentration of flavonoids used was 100 μM.


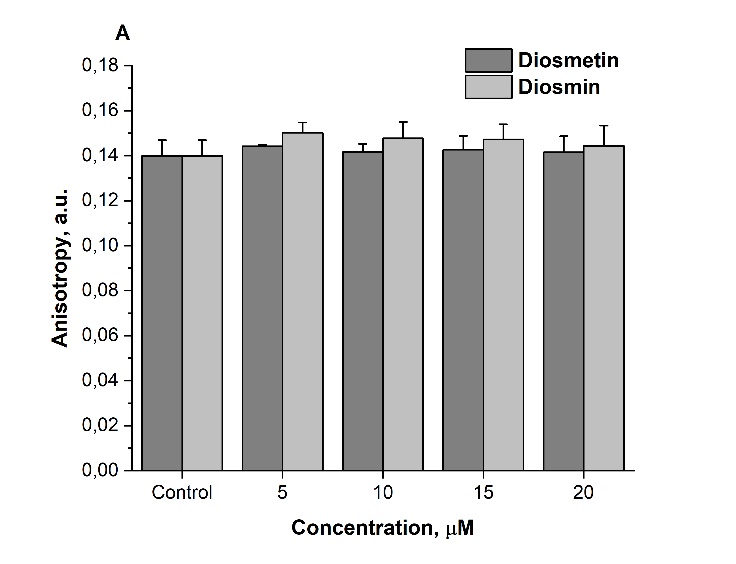

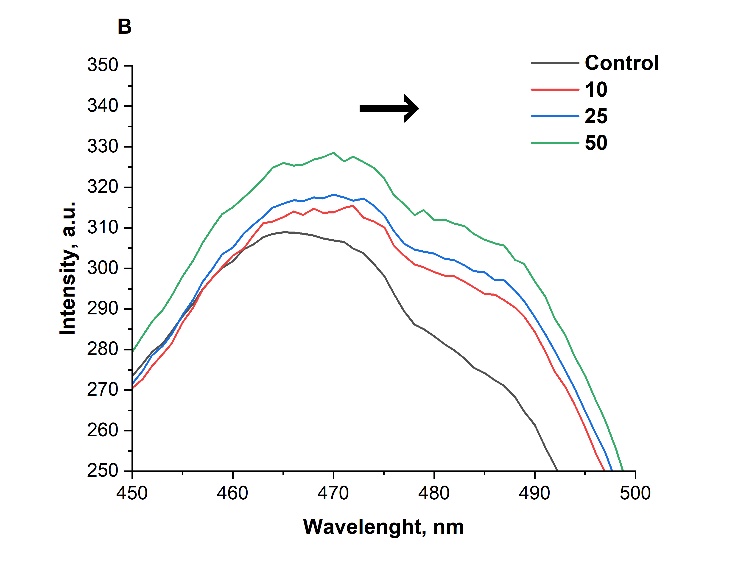


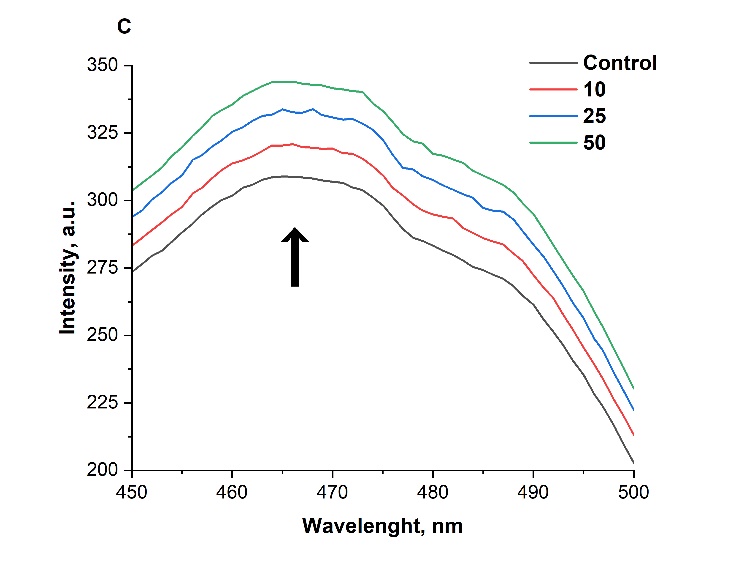


**Supplementary Figure 5.** Interaction of flavonoids with liposomes (DOPC/DOPE/POPS/cholesterol). (A) Changes of DPH anisotropy for liposomes incubated with increasing concentration of diosmetin and diosmin. (B) Fragment of excitation spectra in a range 450-500 nm for di-8-ANEPPS probe with increasing concentration of diosmetin. (C) Fragment of excitation spectra in a range 450-500 nm for di-8-ANEPPS probe with increasing concentration of diosmin. Arrows represents characteristic spectra changes after addition of flavonoids.

**
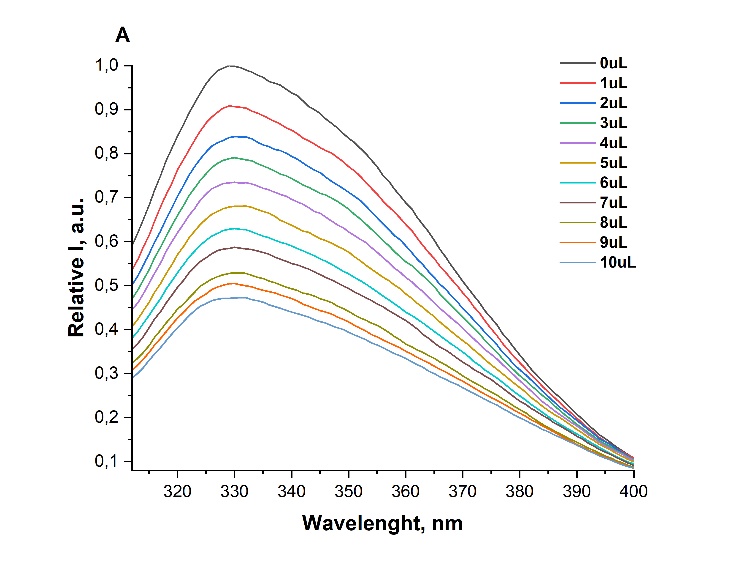

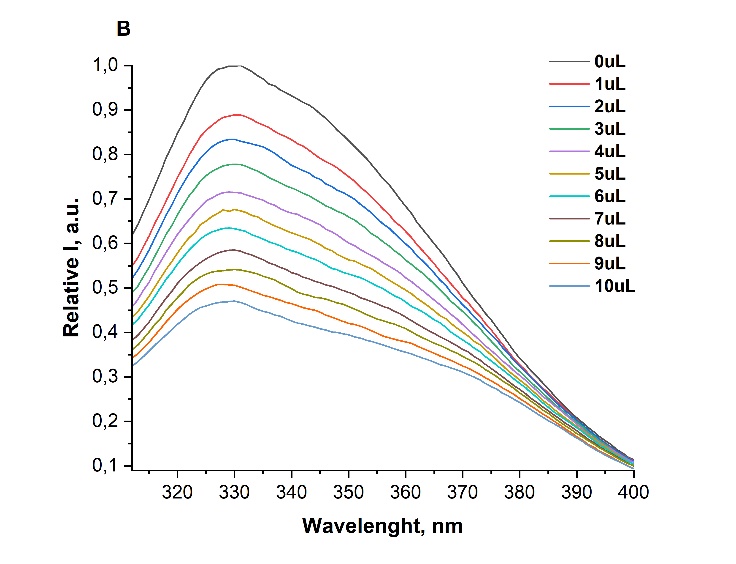
**

**
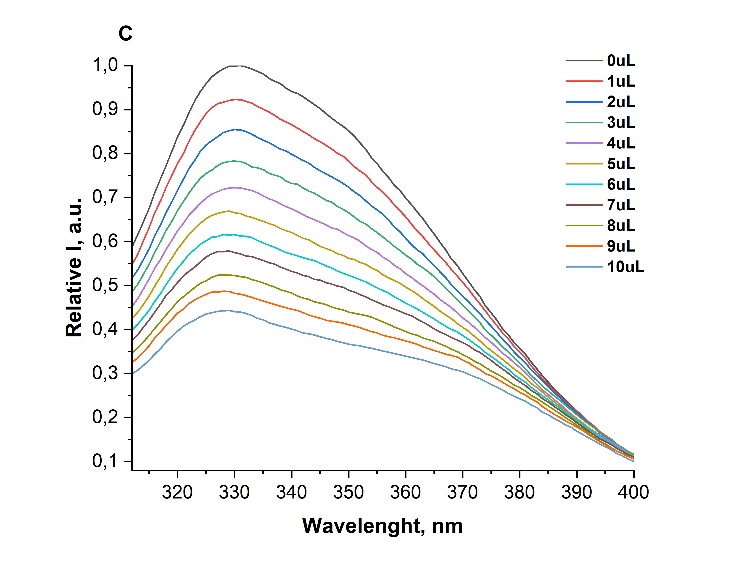
**

**Supplementary Figure 6.** Relative fluorescence spectra between λ=310-400 nm of human hemoglobin (Hb) excited at λ=295 nm. (A) titration of Hb with diosmetin; (B) titration of Hb with diosmin; (C) titration of Hb with diosmetin-7-O-glucoside.


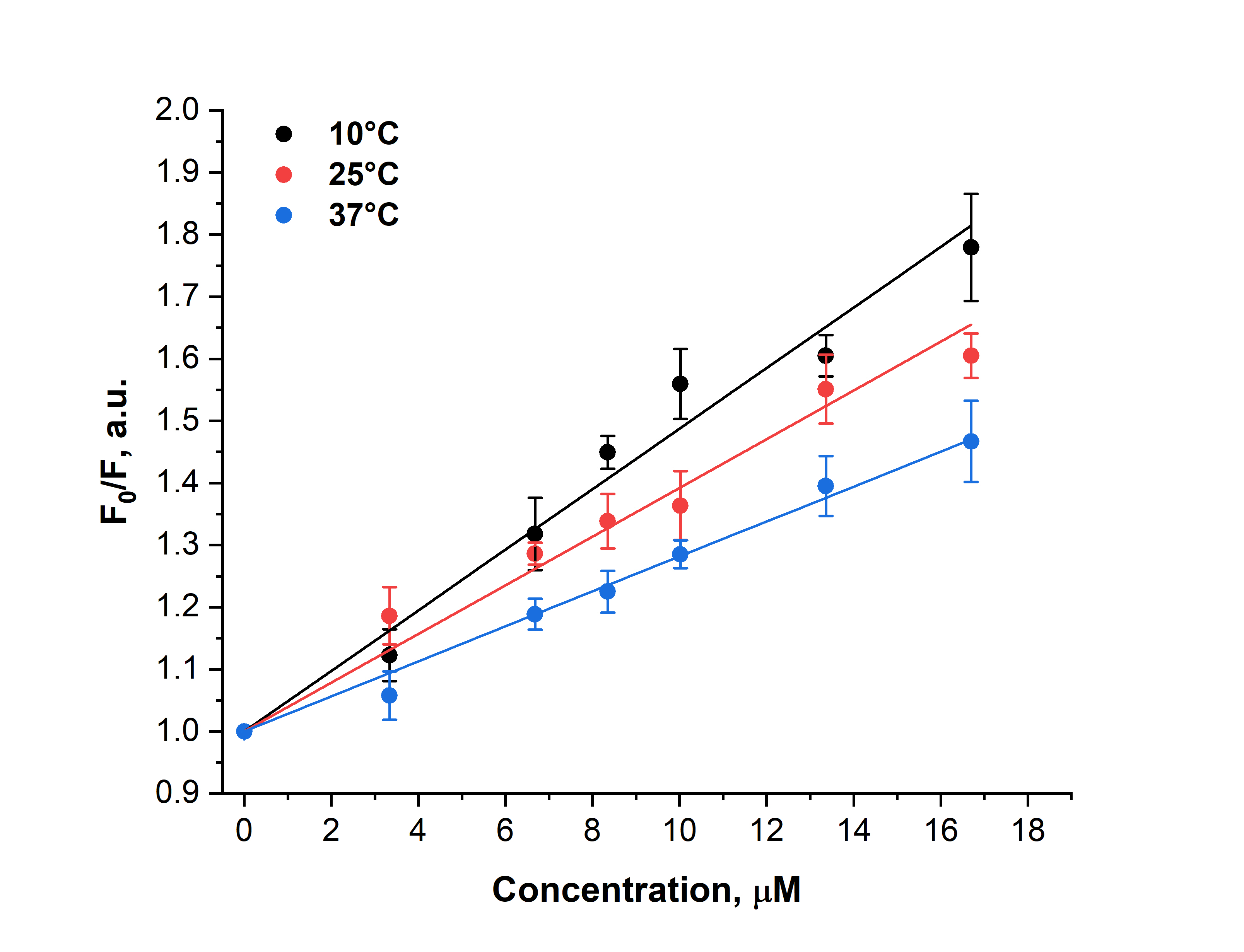


**Supplementary Figure 7.** The dependence of F_0_ /F on the concentration of diosmetin at 10, 25, and 37 °C. The experiment was performed in triplicate. The values are presented as mean ± SD.


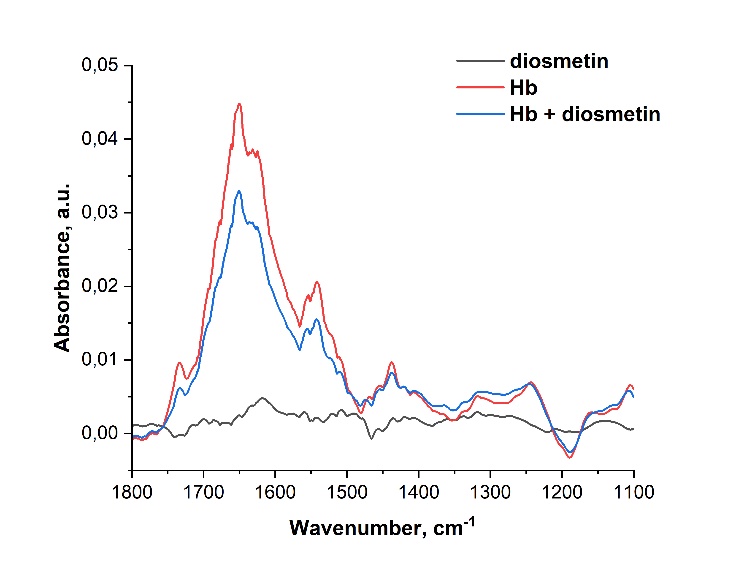


**Supplementary Figure 7.** Validation of background subtraction and diosmetin reference spectrum.

1. **Supplementary text**

Supplementary Figure 7 shows ATR-FTIR spectra of Hb (red), Hb incubated with diosmetin (gray), and free diosmetin (blue), all recorded under identical conditions and processed by subtraction of the phosphate buffer + 2% DMSO spectrum (with optimized scaling, K), followed by identical smoothing and baseline correction. The diosmetin spectrum is included as a reference to indicate ligand-characteristic bands (mainly in the fingerprint region), supporting interpretation of minor differences between Hb and Hb+diosmetin after buffer/DMSO subtraction.
